# Commentary: Differential Risk of Dementia Between Patients With Atrial Flutter and Atrial Fibrillation: A National Cohort Study

**DOI:** 10.3389/fcvm.2022.850968

**Published:** 2022-02-17

**Authors:** Andrea Saglietto, Gaetano Maria De Ferrari, Matteo Anselmino

**Affiliations:** Division of Cardiology, Department of Medical Sciences, “Città della Salute e della Scienza di Torino” Hospital, University of Turin, Turin, Italy

**Keywords:** atrial fibrillation, atrial flutter, dementia, cognitive decline, RR interval irregularity

Atrial fibrillation and atrial flutter: cousin arrhythmias, but non that close. The recent article by Wang et al. (1) provides unprecedented insights on an issue that the scientific community overly takes for granted. Since decades, atrial fibrillation (AF) and atrial flutter (AFL) have been considered as different manifestation of the same disease, and practical guidelines recommend a similar management, in terms of oral anticoagulation prescription, for both AF and AFL (2). However, the pathophysiological background and the clinical presentation of the two conditions are different, particularly related to the ventricular response, completely irregular (“irregularly irregular”) during AF, while generally regular (or, at least, a “regular/modular irregularity”) during AFL. Based on this profound hemodynamic difference, there is a strong scientific rationale that the two “cousin” arrhythmias might present relevant distinctions. Indeed, it was recently suggested that a differential risk in hard clinical endpoints truly exists, with AF presenting a 63, 70, and 8% increased risk of ischemic stroke, heart failure hospitalization and mortality, respectively, if compared to patients with AFL only (3). In his recent article (1), Wang extends the spectrum of clinical differences between AF and AFL. On two propensity-matched cohorts from Taiwan's National Health Insurance Research Database and another dataset (study period 2001–2013), AF relates to an increased risk of dementia compared to AFL, independently from oral anticoagulation (hazard ratio, HR 1.14, 95% CI 1.04–1.25 in patients without oral anticoagulation, and 1.57, 95% CI 1.00–2.45 in patients on warfarin therapy). If on one hand this increased risk of dementia can be partly explained by an increased propension of AF patients to suffer an ischemic stroke, both in non-anticoagulated (HR 1.76, 95% CI 1.56–1.98) and anticoagulated subjects (HR 2.54, 95% CI 1.56–4.12), other mechanisms might certainly be involved.

AF patients are known to be more susceptible to subclinical cardiogenic microembolic phenomena leading to silent cerebral ischemias (4), likely not completely preventable by oral anticoagulation therapy. In addition, a critical role might be played by the rhythm itself, considering the irregularly irregularity of AF, compared to the more regular ventricular response in AFL. In fact, recent evidences point toward a critical role of AF rhythm *per se* on cerebral hemodynamics:

-Reduced mean cerebral blood flow: Gardsdottir et al. (5) demonstrated, at phase contrast MRI, that cerebral blood flow in patients with persistent AF is reduced by about 10%, compared to both paroxysmal AF patients (in sinus rhythm at the time of the test) and controls. In a subsequent analysis, persistent AF patients undergoing elective electrical cardioversion, showed, in case of successful and sustained restoration of sinus rhythm 10 weeks later, an improved cerebral blood flow; no difference could be found, instead, in patients with unsuccessful cardioversion (6). AF, in addition, likely triggers a cerebrovascular hemodynamic dysfunction. The RR variability-induced turbulent blood flow and shear stress pattern alterations induce reduced nitric oxide bioavailability and endothelial dysfunction. Impaired autoregulation and a blunted neurovascular coupling, ultimately result in a chronic reduction of mean cerebral blood flow during ongoing arrhythmia (7).

-Extreme hemodynamic events: AF-related beat-to-beat variability, assessed by spatially resolved near-infrared spectroscopy, alters cerebral microvascular perfusion by inducing transient and repetitive hypoperfusions or hypertensive events in the deep cerebral circle. Interestingly, while these events disappear after sinus rhythm restoration by electrical cardioversion, the same does not occur in a comparable subgroup of AFL patients, supporting the hypothesis that a more regular ventricular response results less impacting on the deep cerebral circle (8).

Altogether these rhythm-induced hemodynamic mechanisms (decreased mean cerebral blood flow with superimposed transient extreme hemodynamic events in the deep cerebral circle) might concur in the process of progressive cerebral damage differently leading to dementia in AF and AFL patients (Figure 1).

**Figure 1:**
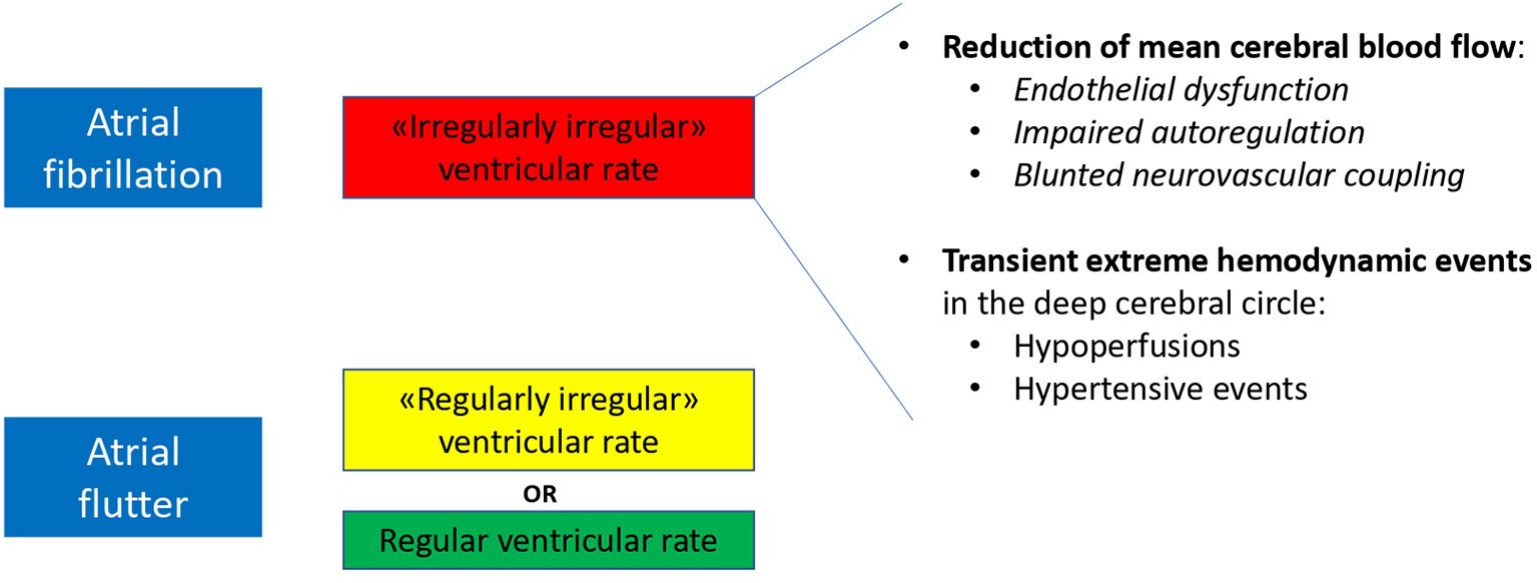
Proposed hemodynamic determinants of a differential risk of dementia in patients with AF and AFL.

## Author Contributions

AS conceived the commentary. All authors contributed in manuscript writing.

## Conflict of Interest

The authors declare that the research was conducted in the absence of any commercial or financial relationships that could be construed as a potential conflict of interest.

## Publisher's Note

All claims expressed in this article are solely those of the authors and do not necessarily represent those of their affiliated organizations, or those of the publisher, the editors and the reviewers. Any product that may be evaluated in this article, or claim that may be made by its manufacturer, is not guaranteed or endorsed by the publisher.
